# Local Dynamic Stability of Trunk During Gait is Responsive to Rehabilitation in Subjects with Primary Degenerative Cerebellar Ataxia

**DOI:** 10.1007/s12311-024-01663-4

**Published:** 2024-01-27

**Authors:** Stefano Filippo Castiglia, Dante Trabassi, Carmela Conte, Valeria Gioiosa, Gabriele Sebastianelli, Chiara Abagnale, Alberto Ranavolo, Cherubino Di Lorenzo, Gianluca Coppola, Carlo Casali, Mariano Serrao

**Affiliations:** 1 Department of Medico-Surgical Sciences and Biotechnologies, “Sapienza” University of Rome-Polo Pontino, Corso Della Repubblica 79, 04100 Latina, Italy; 2 Department of Brain and Behavioral Sciences, University of Pavia, 27100 Pavia, Italy; 3 Department of Occupational and Environmental Medicine, Epidemiology and Hygiene, INAIL, Via Fontana Candida, 1, Monte Porzio Catone, 00078 Rome, Italy; 4 Movement Analysis Laboratory, Policlinico Italia, Piazza del Campidano, 6, 00162 Rome, Italy

**Keywords:** Gait Analysis, Rehabilitation, Postural Balance, Cerebellar Ataxia, Gait Stability, Inertial Measurement Units

## Abstract

This study aimed to assess the responsiveness to the rehabilitation of three trunk acceleration-derived gait indexes, namely the harmonic ratio (HR), the short-term longest Lyapunov’s exponent (sLLE), and the step-to-step coefficient of variation (CV), in a sample of subjects with primary degenerative cerebellar ataxia (swCA), and investigate the correlations between their improvements (∆), clinical characteristics, and spatio-temporal and kinematic gait features. The trunk acceleration patterns in the antero-posterior (AP), medio-lateral (ML), and vertical (V) directions during gait of 21 swCA were recorded using a magneto-inertial measurement unit placed at the lower back before (T0) and after (T1) a period of inpatient rehabilitation. For comparison, a sample of 21 age- and gait speed-matched healthy subjects (HS_matched_) was also included. At T1, sLLE in the AP (sLLE_AP_) and ML (sLLE_ML_) directions significantly improved with moderate to large effect sizes, as well as SARA scores, stride length, and pelvic rotation. sLLE_ML_ and pelvic rotation also approached the HS_matched_ values at T1, suggesting a normalization of the parameter. HRs and CV did not significantly modify after rehabilitation. ∆sLLE_ML_ correlated with ∆ of the gait subscore of the SARA scale (SARA_GAIT_) and ∆stride length and ∆sLLE_AP_ correlated with ∆pelvic rotation and ∆SARA_GAIT_. The minimal clinically important differences for sLLE_ML_ and sLLE_AP_ were ≥ 36.16% and ≥ 28.19%, respectively, as the minimal score reflects a clinical improvement in SARA scores. When using inertial measurement units, sLLE_AP_ and sLLE_ML_ can be considered responsive outcome measures for assessing the effectiveness of rehabilitation on trunk stability during walking in swCA.

**Supplementary Information:**

The online version contains supplementary material available at 10.1007/s12311-024-01663-4.

## Introduction

Due to the cerebellum’s inability to process multisensory features and provide adequate computation and corrections to perturbations [1–5], subjects with degenerative cerebellar ataxia (swCA) exhibit poor joint coordination, abnormal intra-limb joint, and upper and lower body segment coupling during walking [5–9]. Ataxic gait is additionally characterized by incoordination between the upper and lower bodies, which results in increased upper body oscillations with a lack of local trunk stability, transforming the trunk into a generator of perturbations during walking. This causes an unstable, staggering, and wide-based gait, which correlates with disease progression and leads to impaired balance and an increased risk of falling [10–15].

SwCA attempts to cope for center of mass deviations by increasing the base of support and coactivating muscles at single and multi-joint levels, in order to stiffen the lower limb joints [16–18]. Nonetheless, approximately 85% of swCA experience injurious falls. Intensive and repetitive rehabilitation focusing on balance, gait, and activities of daily living has proved to be effective in improving motor performance of swCA [19–27]. Particularly, truncal ataxia and trunk-limb coordination were shown to be effective targets for rehabilitation [19, 20, 22, 28]. Therefore, identifying responsive measures to quantify the improvements in trunk instability during gait may provide clinicians with useful information for designing rehabilitative interventions and assessing their effectiveness.

In this regard, instrumented gait analysis is a useful tool for capturing gait abnormalities and improvements after interventions in swCA, providing specific measures that outperform traditional clinical assessment tools in terms of accuracy and sensitivity to changes and allowing separate investigation of several aspects of gait ataxia [29–32].

Recently, a series of trunk acceleration-derived gait measures have been proposed to assess gait imbalance [29, 33–43]. Particularly, three indexes, namely the harmonic ratios (HRs), short-time longest Lyapunov’s exponent (sLLE), and step-to-step coefficient of variation (CV), showed the best ability to characterize the trunk behavior during gait of swCA [36–38].

Therefore, the primary aim of this study was to assess the internal responsiveness to rehabilitation of HRs, sLLE, and CV in terms of the magnitude of changes after rehabilitation. Furthermore, we aimed to assess (i) the external responsiveness of the gait stability indexes, as well as the minimal clinically important differences reflecting clinical improvements after rehabilitation, and (ii) the spatio-temporal and kinematic gait parameters that correlate with the improvements in the gait stability indexes and clinical improvements following rehabilitation in swCA.

We hypothesized that, in addition to characterizing swCA gait behavior, HRs, sLLE, and CV could be responsive outcomes for rehabilitative interventions, and that their improvements could correlate with clinical improvements after rehabilitation.

## Materials and Methods

### Subjects

We collected data samples from 21 swCA (8 females, 13 males) aged 51.33 ± 12.17 years and diagnosed with primary degenerative cerebellar ataxia since 10.71 ± 7.14 years, who underwent an inpatient rehabilitation program at the Traumatic Orthopedic Surgical Institute (ICOT) in Latina, Italy. Table 1 shows the diagnoses and clinical characteristics of all subjects. The Scale for the Assessment and Rating of Ataxia (SARA) [44, 45] and its gait subscore (SARA_GAIT_) were administered to assess disease severity. SwCA with gait impairment due to extracerebellar symptoms (spasticity, polyneuropathy, cognitive impairment (MMSE score > 24), oculomotor abnormalities, and visual deficits according to the Snellen visual acuity test) was excluded, as well as subjects with concomitant other neurologic or orthopedic conditions. We only included subjects who could walk without assistance and had gait problems that were exclusively cerebellar in nature at the time of their initial evaluation within a larger group of swCA from a rare disease center [13, 18, 36, 46].

**Table 1:** Sample characteristics and gait assessments at T0 and T1

		swCA	T0 (mean (SD))	T1 (mean (SD))	*p*-value (T0 vs T1)	Cohen’s *d*	HS
Disease duration (years)		10.71 (7.14)					
CA subtype (*n*)	SAOA	10					
	SCA1	5					
	SCA2	2					
	SCA3	2					
	SCA6	1					
	SCA8	1					
SARA (total)			14.74 (7.84)	9.69 (3.95)	0.01	0.66	
SARA_GAIT_			3.09 (0.99)	2.43 (1.03)	0.00 •	0.73	
Age (years)			51.33 (12.17)			56.61 (10.92)	51.33 (12.17)
Gait speed (m/s)			1.04 (0.25)	1.02 (0.25)	0.17 •	0.31	1.01 (0.15)
HR_AP_			1.86 * (0.37)	1.85 * (0.44)	0.89	0.03	2.51 (0.53)
HR_ML_			1.85 * (0.39)	1.81 * (0.42)	0.58	0.12	2.44 (0.44)
HR_V_			1.90 * (0.43)	1.89 * (0.50)	0.96	0.01	2.81 (0.57)
sLLE_AP_			0.95 * (0.29)	0.75 * (0.21)	0.03	0.75	0.55 (0.26) †
sLLE_ML_			0.61 * (0.18)	0.41 (0.13)	0.00	0.86	0.34 (0.16) †
sLLEv			0.62 * (0.31)	0.49 * (0.19)	0.06	0.43	0.33 (0.20) †
Step-to-step CV (%)			39.73 * (20.42)	42.58 * (19.00)	0.38	0.22	18.55 (11.45) †
Stride length (m)			1.14 * (0.23)	1.19 * (0.25)	0.04	0.46	1.27 (0.19)
Stance phase duration (% gait cycle)			65.42 * (3.45)	63.98 * (3.53)	0.11	0.36	62.11 (4.38) †
Swing phase duration (% gait cycle)			34.70 * (3.26)	36.02 * (3.53)	0.12	0.35	37.89 (4.38) †
Double support phase duration (% gait cycle)			30.16 * (7.21)	28.38 * (6.99)	0.22	0.27	23.49 (6.75)
Single support phase (% gait cycle)			35.26 * (3.92)	35.61 * (4.16)	0.67	0.09	38.62 (3.47)
Cadence (steps/min)			107.63 (12.12)	105.72 (9.22)	0.44	0.17	101.82 (12.52) †
Pelvic tilt (°)			2.11 (0.82)	2.19 (0.93)	0.63	0.10	2.90 (1.02)
Pelvic obliquity (°)			1.97 * (1.11)	2.47 * (1.47)	0.07 •	0.48	5.19 (2.65)
Pelvic rotation (°)			5.84 * (2.65)	7.84 (4.63)	0.03	0.49	8.12 (3.11) †

*swCA*, subjects with degenerative cerebellar ataxia; *HS*, age- and gait speed-matched healthy subjects; *SAOA*, sporadic adult-onset ataxia; *SCA*, spino-cerebellar ataxia; SARA, scale for the assessment and rating of ataxia; *SARA*_*GAIT*_, gait subscore of the SARA scale; *HR*, harmonic ratio; *sLLE*, short-term largest Lyapunov’s exponent; *AP*, antero-posterior direction; *ML*, medio-lateral direction; *V*, vertical direction; *CV*, coefficient of variation; •, *p*-values calculated using Wilcoxon test following significant Shapiro–Wilk test (SARA_GAIT_, *p* = 0.01; gait speed, *p* = 0.01; pelvic obliquity, *p* = 0.03); †, *p*-values calculated using Mann–Whitney test following significant Shapiro–Wilk test in HS subgroup (sLLE_AP_, *p* = 0.00; sLLE_ML_, *p* = 0.00; sLLE_V_, *p* = 0.04; step-to-step CV, *p* = 0.01; stance phase duration, *p* = 0.02; swing phase duration, *p* = 0.02; cadence, *p* = 0.00; pelvic rotation, *p* = 0.01); *significant differences between swCA and HS values

SwCA were matched with a dataset of 89 walking trials from healthy subjects (HS) for group comparison using a 1:1 optimal data matching procedure with the propensity score difference method [47]. Each HS repeated the gait task twice, walking at both self-selected and slower speeds to reduce the effect of gait speed on other gait parameters and to gather the largest sample size possible for speed-matched comparisons [18, 34, 36, 48]. The propensity scores were calculated using logistic regression analysis and age and speed as covariates [49–53]. As a control group, 21 age- and speed-matched healthy subjects (HS_matched_) (11 females, 10 males), aged 56.61 ± 10.92 years, were included after the matching procedure. An independent sample *t*-test confirmed the effectiveness of the matching procedure (Table 1).

Before the experimental procedure, both swCA and HS provided informed consent in accordance with the Helsinki Declaration. The local ethics committee (CE Lazio 2, protocol number 0139696/2021) approved the study.

### Procedures

Gait data were collected by fixing a magneto-inertial measurement unit (BTS GWALK, BTS, Milan, Italy) at the fifth lumbar vertebrae level via an ergonomic belt and connecting it to a laptop via Bluetooth. The “Walk + ” protocol of the G-STUDIO software (G-STUDIO, BTS, Milan, Italy) was used to detect trunk linear acceleration patterns in the antero-posterior (AP), medio-lateral (ML), and vertical (V) directions at a sampling rate of 100 Hz, as well as to detect right and left heel strikes, toe-off, spatio-temporal parameters, and pelvic kinematics. swCA were asked to walk at their own pace along a corridor 30 m long and 3 m wide with no external sensory cues before (T0) and at the end of their 4-week rehabilitation period (T1). Walking trials with at least 20 consecutive correctly recorded strides [54–56] were included in the analyses. HRs, CV, and sLLE in the three spatial directions were also calculated as the trunk acceleration-derived gait indexes. Details on the calculation of the indexes are provided in the supplementary material.

The rehabilitation program consisted of a 4-week inpatient intensive training. All subjects received 3 h of day rehabilitation per day, with at least 1 h of exercise-based rehabilitation 6 days a week, for a total of 18 h and 6 h of exercise therapy per week. Based on the talk test, the exercises were performed at a moderate intensity. Exercises focused on static balance and trunk stabilization, dynamic balance, trunk-limb coordination, and stretching exercises to treat or prevent contractures [19, 20, 22, 26]. Static balance exercises were performed using both “hands-off” (e.g., standing on one leg or maintaining specific posture such as side bridging) and “hands-on” interventions (e.g., rhythmic stabilization of the trunk in quadruped, supine, lateral position, and half-kneeling positions); dynamic balance exercises were performed through the repetitive execution of postural transfers from lying to standing positions; trunk-limb coordination was trained by asking participants to move their upper or lower limbs alternatively while stabilizing the trunk in supine (e.g., raising feet alternatively while maintaining supine bridge position), lateral (e.g., raising one leg or one arm while maintaining a stable side-lying position), prone (e.g., raising arms or legs alternatively while maintaining quadruped position), or vertical (e.g., moving feet forward alternatively while maintaining kneeling position) positions. Participants also performed exercises aiming at reducing their postural sway by using an instrumented balance board with visual feedback (Tecnobody, Prokin Balance System, Tecnobody, Italy).

### Statistical Analysis

Based on previously reported gait improvements after rehabilitation [20], we calculated a sample size of at least 15 swCA to reliably detect a high effect size (*d* = 0.8), assuming a two-sided detection criterion that allows for a maximum type I error rate of 0.05 and type II error rate of 0.80 in a paired sample *t*-test procedure.

The Shapiro–Wilk test was used to check for the normality of the distributions. To identify significant changes in clinical and gait parameters at T1, a paired *t*-test or Wilcoxon test was used. Cohen’s *d* with Hedge’s correction was used to calculate internal responsiveness [57, 58].

To identify significant differences between swCA and HS at T0 and the gait parameters that approached normative values at T1, the unpaired *t*-test or Mann–Whitney test was used.

Gait variable and SARA and SARA_PG_ score changes at T1 were expressed as delta (∆) values using the following formula:

$$\Delta =100\times \frac{{{\text{value}}}_{{\text{T}}1}- {{\text{value}}}_{{\text{T}}0}}{{{\text{value}}}_{{\text{T}}0}}$$

To identify the correlations between the ∆*s* of the modified gait stability indexes and the ∆*s* of the clinical, spatio-temporal, and kinematic gait parameters, Spearman’s correlation coefficients were calculated. To account for tied scores, we applied the tie correction factor to the correlation coefficients according to the formula:

$${r}_{{\text{s}}}=1- \frac{6\sum {d}_{i}^{2}}{n\left({n}^{2}-1\right)- \sum \left({t}_{{\text{i}}}^{3}-{t}_{{\text{i}}}\right)},$$

where *d*_i_ represents the difference between the ranks of corresponding values for the two variables, *n* is the number of observations, and *t*_i_ is the number of tied ranks for each group of ties. The term $\sum \left({t}_{{\text{i}}}^{3}-{t}_{{\text{i}}}\right)$ represents the sum of the cubes of the number of ties minus the number of ties for each distinct number of tied values, summed across all sets of ties.

The external responsiveness of the modified trunk acceleration-derived indexes was assessed using an anchor-based method [57] using the smallest detectable change of the SARA scale (3.5 points) in subjects who improved their SARA_GAIT_ scores as the criterion for clinical improvement [59]. Receiver operating characteristic curves were plotted and the area under the curves (AUCs) were calculated. AUC values > 0.70 were considered for satisfying external responsiveness. The value that maximized the sum of sensitivity and specificity was used to determine the minimally clinically important differences (MCID). At the MCID value, positive and negative likelihood ratios (LRs) were calculated and transformed into post-test probabilities using Fagan’s nomograms. ∆*s* of the externally responsive trunk acceleration-derived gait indexes was classified as the binary variable based on their MCIDs, and ∆SARA_GAIT_ was categorized as lacking improvement (∆SARA_GAIT_ < 1), improving by 1 point (∆SARA_GAIT_ ≥ 1), or improving by 2 points or higher (∆SARA_GAIT_ ≥ 2). To observe differences in baseline variables and improvements after rehabilitation using the MCID-based category as a between-groups factor, a Mann–Whitney test for continuous independent variables was used.

All the statistical analyses were set at 95% significance level and 80% power and performed using the IBM SPSS vers.27, JASP vers.0.17.2.1, NCSS 2023 software, and the “Pingouin” Python package, vers.0.5.3 [60].

## Results

### Internal Responsiveness Findings

The results of the clinical and gait parameter assessments at T0 and T1 are reported in Table 1.

**Fig. 1:**
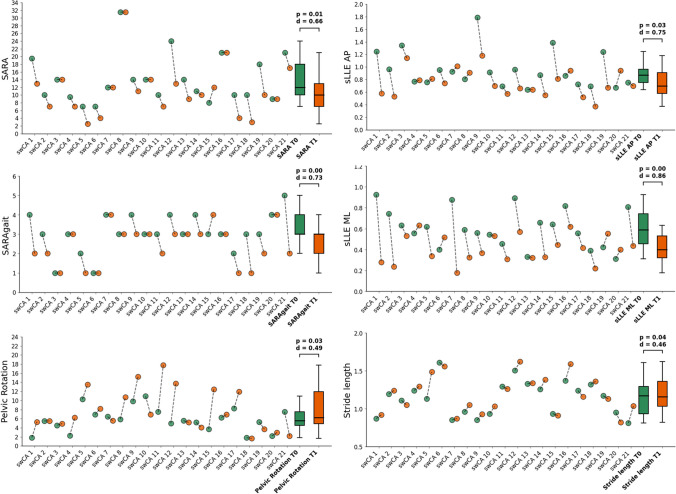
Significant differences between T0 (green) and T1 (orange). The jitter elements represent the values for each subject at T0 and T1. Paired sample *t*-test was performed on SARA, sLLE_AP_, sLLE_ML_, and pelvic rotation after verifying the normality of distributions (Shapiro–Wilk test, *p* > 0.05). Wilcoxon test was performed on SARA_GAIT_ (Shapiro–Wilk test, *p* = 0.01). Above the boxplots, Cohen’s *d* as the effect size and *p*-values

**Fig. 2:**
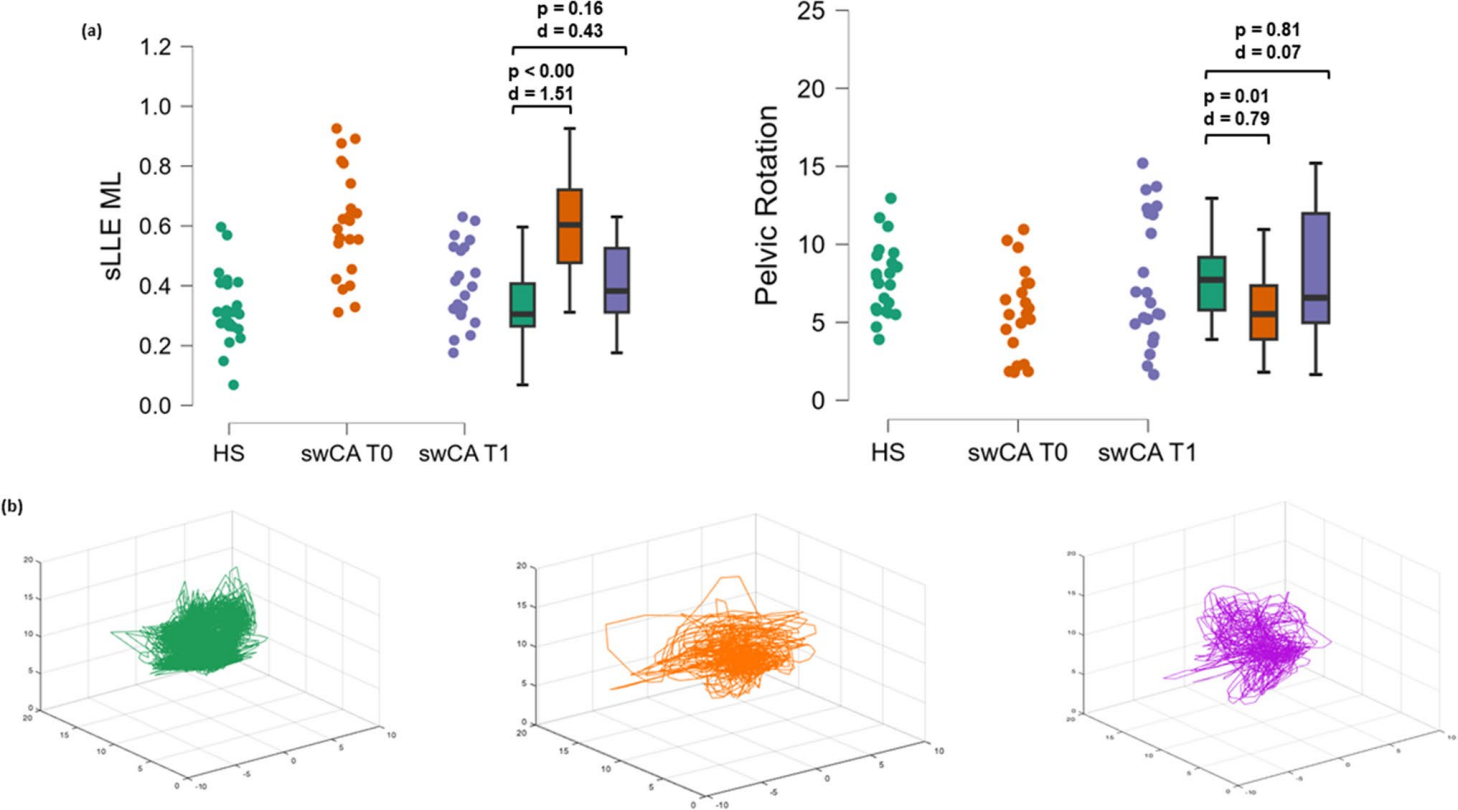
**a** Differences in short-term longest Lyapunov’s exponent (sLLE) in the medio-lateral (ML) direction, and pelvic rotation between HS (green) and swCA at T0 (orange) and swCA at T1 (purple). Cohen’s *d* as the effect size and *p*-values in a *t*-test or Wilcoxon test procedure are reported. **b** 3D-reconstructed state space of the acceleration and its time-delayed copies (time delay of ten data samples) in the medio-lateral direction of a representative age- and speed-matched healthy subject (green), and a swCA at baseline (orange) and after the rehabilitation period (purple)

At T0, significant differences between swCA and HS were found in all the gait parameters except for gait speed and pelvic tilt. Following rehabilitation, swCA significantly improved their SARA and SARA_GAIT_ scores, stride length, pelvic rotation, and sLLE_AP_ with medium-to-large effect sizes and sLLE_ML_ with large effect size (Table 1, Fig. 1). At T1, sLLE_ML_ and pelvic rotation were no longer different from HS_matched_ (Fig. 2).

### Correlation Findings

Both ∆sLLE_ML_ and ∆sLLE_AP_ correlated with ∆SARA_GAIT_. ∆sLLE_ML_ correlated with ∆stride length, whereas ∆sLLE_AP_ correlated with ∆pelvic rotation (Table 2).

**Table 2:** Correlation analysis findings

		∆SARA_GAIT_^*^	∆Pelvic rotation	∆Stride length
∆sLLE_ML_	*ρ*	0.41	0.18	0.51
	*p*-value	0.03	0.22	0.01
∆sLLE_AP_	*ρ*	0.43	0.41	− 0.07
	*p*-value	0.03	0.03	0.62

∆, %difference between the last and the first assessment; *sLLE*, short-term largest Lyapunov’s exponent; *AP*, antero-posterior direction; *ML*, medio-lateral direction; *SARA*_*GAIT*_, subscore of the SARA scale, *ρ*, Spearman’s correlation coefficient; *, Spearman’s rho coefficients accounting for tied scores

### External Responsiveness Findings

Nine swCA (40.91%) outperformed the smallest detectable SARA change score among the 11 (52.38%) swCA who improved their SARA_GAIT_ score by 1 point or more. Both sLLE_ML_ and sLLE_AP_ revealed good external responsiveness to SARA improvements (AUCs ≥ 0.70). According to MCID, ∆sLLE_ML_ ≥ 36.16% and ∆sLLE_AP_ ≥ 28.19% were deemed as necessary to improve SARA scores with 70% and 67% probabilities, respectively (Table 3).

**Table 3:** External responsiveness findings

	AUC	MCID	Se	Sp	LR +	LR −	PTP +	PTP −
∆sLLE_ML_	0.75 (0.41–0.91)	≥ 36.16%	0.78	0.77	3.37	0.28	70%	16%
∆sLLE_AP_	0.70 (0.36–0.88)	≥ 28.19%	0.66	0.77	2.89	0.43	67%	23%

*AUC*, area under the ROC curve; *MCID*, minimal clinically important difference; *Se*, sensitivity; *Sp*, specificity; *LR* + , positive likelihood ratio; *LR* − , negative likelihood ratio; *PTP* + , positive post-test probability; *PTP* − , negative post-test probability; *∆sLLE*, percentage improvement of short-term longest Lyapunov’s exponent following rehabilitation; *ML*, medio-lateral direction of the acceleration signals; *AP*, antero-posterior direction of the acceleration signals

Twelve (57.14%) and 11 (52.38%) swCA were classified as improved according to the MCIDs of sLLE_ML_ and sLLE_AP_, respectively. We found significant differences in sLLE_ML_ baseline values, and ∆stride length, based on sLLE_ML_ improvements, and in sLLE_AP_ baseline values and ∆pelvic rotation, based on sLLE_AP_ improvements (Table 4). Three subjects (14.29%) improved both their SARA_GAIT_ values and sLLE values. After removing these subjects from the analysis, no differences in ∆SARA_GAIT_ were found based on sLLE_ML_ and sLLE_AP_ improvements (Table 4). Significant differences between T0 and T1 were maintained in sLLE_ML_ and sLLE_AP_ even after repeating a paired sample *t*-test by removing swCA with ∆SARA_GAIT_ > 1 (sLLE_ML_, *p* = 0.00, Cohen’s *d* = 0.76; sLLE_AP_, *p* = 0.02, Cohen’s *d* = 0.60).

**Table 4:** Significant differences according to MCIDs

		∆sLLE_ML_ ≥ 36.16%	∆sLLE_ML_ < 36.16%	*p*-value	∆sLLE_AP_ ≥ 28.19%	∆sLLE_AP_ < 28.19%	*p*-value
SARA_GAIT_ T0 (*n*, %)	1	2 (9.52)	0		0	2 (9.52)	
	2	1 (4.76)	1 (4.76)		1 (4.76)	1 (4.76)	
	3	7 (33.33)	3 (14.29)		5 (23.81)	5 (23.81)	
	4	2 (9.52)	5 (23.81)		5 (23.81)	2 (9.52)	
SARA T0 (mean (SD))		16.22 (8.18)	12.37 (4.22)	0.30	14.50 (5.34)	13.50 (7.57)	0.37
∆SARA (%, mean (SD))		33.92 (26.59)	17.56 (29.45)	0.20	28.82 (33.59)	19.92 (23.26)	0.24
SARA_GAIT_ T0 (mean (SD))		3.44 (0.73)	2.75 (0.96)	0.08	3.36 (0.67)	2.70 (1.06)	0.24
∆SARA_GAIT_ (*n*, %)	< 1	2 (9.52)	8 (38.10)		2 (9.52)	8 (38.10)	
	≥ 1	4 (19.05)	4 (19.05)		6 (28.57)	2 (9.52)	
	≥ 2	3 (14.29)	0		3 (14.29)	0	
∆SARA_GAIT_ (%, mean (SD))		34.44 (24.31)	9.03 (22.32)	**0.03**	30.45 (28.37)	8.33 (18.00)	**0.04**
∆SARA_GAIT_* (%, mean (SD))		22.22 (19.48)	9.03 (22.32)	0.27	19.79 (25.56)	8.33 (18.00)	0.56
sLLE_AP_ T0 (mean (SD))		0.88 (0.17)	0.99 (0.36)	**0.02**	1.11 (0.35)	0.84 (0.20)	**0.03**
sLLE_ML_ T0 (mean (SD))		0.72 (0.17)	0.52 (0.14)	**0.01**	0.65 (0.18)	0.56 (0.19)	0.28
Gait speed T0 (m/s, mean (SD))		1.08 (0.21)	1.02 (0.28)	0.57	1.07 (0.27)	1.03 (0.24)	0.75
Stride length (m, mean (SD))		1.09 (0.24)	1.17 (0.22)	0.52	1.18 (0.23)	1.01 (0.23)	0.34
∆Stride length (%, mean (SD))		11.27 (10.78)	0.28 (8.33)	**0.02**	5.94 (9.26)	3.95 (12.63)	0.47
Stance phase (%gait cycle, mean (SD))		65.53 (4.74)	65.35 (2.29)	0.97	64.65 (2.94)	66.12 (3.86)	0.47
Swing phase (%gait cycle, mean (SD))		34.77 (4.39)	34.65 (2.29)	0.97	34.12 (3.56)	35.34 (2.94)	0.47
Double support phase (%gait cycle, mean (SD))		30.81 (10.03)	29.67 (4.56)	0.73	28.29 (6.01)	31.86 (8.05)	0.39
Single support phase (%gait cycle, mean (SD))		34.72 (5.32)	35.67 (2.64)	0.59	34.26 (4.30)	36.36 (3.34)	0.35
Cadence (steps/min, mean (SD))		113.41 (10.40)	103.30 (11.85)	0.06	108.19 (13.28)	107.02 (11.38)	0.86
Pelvic obliquity (°, mean (SD))		2.05 (1.12)	1.92 (1.16)	0.78	1.92 (0.89)	2.04 (1.37)	0.86
Pelvic rotation (°, mean (SD))		5.49 (2.62)	6.10 (2.75)	0.57	5.89 (2.98)	5.78 (2.39)	0.78
∆Pelvic rotation (%, mean (SD))		30.77 (75.45)	0.81 (59.64)	0.32	39.49 (48.61)	− 11.38 (58.23)	**0.02**

∆, differences between the assessment before and after rehabilitation; *SARA*, scale for the assessment and rating of ataxia; *SARA*_*GAIT*_, gait subscore of the SARA scale; *sLLE*, short-term largest Lyapunov’s exponent; *AP*, antero-posterior direction; *ML*, medio-lateral direction; *p*-values were calculated using Mann–Whitney test; *, statistical analysis performed by excluding swCA with ∆SARA_GAIT_ > 1; bolded values represent significant differences

## Discussion

The main objective of this study was to assess the responsiveness of seven trunk acceleration-derived gait indexes to intensive rehabilitation training in a sample of swCA by determining the magnitude of their modification and quantifying the minimal improvements required to identify subjects who clinically improved following rehabilitation. We also aimed at identifying significant correlations between the improvements in trunk acceleration-derived gait indexes and the improvements in clinical and kinematic measures.

We found that sLLE in the AP and ML directions significantly modified following rehabilitation with medium-to-high internal (0.75 < *d* > 0.86, Table 1, Fig. 1) and good external (AUCs ≥ 0.70, Table 3) responsiveness. Following rehabilitation, sLLE_ML_ also approached normative values (Fig. 2). Furthermore, ∆sLLE_ML_ and ∆sLLE_AP_ moderately correlated with ∆SARA_GAIT_ (Table 2), with MCIDs ≥ 36.16% and ≥ 28.19%, respectively, required to detect clinically significant variations in SARA scale values after rehabilitation (Table 3).

sLLE quantifies the local dynamic stability of a system [61–63], accurately reflecting the gait instability of swCA caused by the inability to recover from small perturbations [33, 36, 38]. However, to the best of our knowledge, this is the first time that its responsiveness to interventions has been investigated in swCA. Because the acceleration patterns in this study were recorded at the lower back level, and trunk ataxia has been described as being susceptible to improvement in swCA [19, 64], we could argue that sLLE may capture improvements in the ability of the trunk to cope with the center of mass displacements during gait. In that regard, it represents a potentially useful tool to assess the efficacy of intensive rehabilitation treatments. We additionally found that subjects with higher baseline sLLE values improved the most in dynamic trunk stability after rehabilitation (Table 4). Although our findings did not reach statistical significance, it is worth noting that a substantial number of subjects with cerebellar ataxia (swCA) who improved the most in sLLE_ML_, 33.33%, specifically (Table 4), had poorer gait quality at baseline, according to their SARA_GAIT_ scores. This observation is consistent with existing literature [20, 65] reporting the effectiveness of rehabilitation training in swCA in more severely disabled swCA, regardless of disease duration, implying that individuals with swCA may be able to improve their dynamic trunk stability despite ongoing degeneration.

Notably, unlike other studies investigating the effects of rehabilitation on gait parameters [19, 20, 24], gait speed did not improve in our sample, suggesting that sLLE may capture the effects of rehabilitation regardless of gait speed. The differences in gait speed improvements observed in our study compared to existing literature can be attributed to differences in rehabilitation environments, methods of gait speed assessment, and the nature of exercise programs implemented. Miyai et al. [19] and Keller et al. [24] documented increases in gait speed following 4 and 6 weeks of rehabilitation programs, respectively, focusing specifically on gait training. Ilg et al. [20] also observed gait improvements following a 16-week rehabilitation period, observing improvements in gait speed after an 8-week follow-up, and involved participants engaging in home-based exercises following intensive rehabilitation. In contrast, our study involved inpatient swCA and did not emphasize specialized gait training, such as on uneven terrain, outdoor settings, or treadmill training with variable gait speeds. This could partly explain the lack of significant gait improvement findings. However, a direct comparison with Miyai et al. is challenging, as they did not specify the tools used for gait speed assessment or the distance for gait measurement. Keller et al. [24] employed an optoelectronic system to measure gait over a 6-m path, significantly shorter than the 30-m distance that we used in our study, leading to the recording of a considerably fewer number of consecutive gait strides. Ilg et al. [20], on the other hand, measured gait parameters over 12–15 strides using an optoelectronic system. Their study found that increased walking speeds were achieved after an 8-week period of home exercises following 4 weeks of supervised intensive rehabilitation, resulting in a considerably longer observation period compared with our study. Thus, the disparities in our study’s findings could be attributed to the different lengths and types of rehabilitation programs.

As in previous studies on swCA rehabilitation [20], we also found significant modifications in stride length (Table 1, Fig. 1) that positively correlated with the improvements in sLLE_ML_ (Table 2). Moreover, stride length improvements were significantly higher in swCA who improved their sLLE_ML_ by the 36.16% MCID (Table 4). Because of the use of a single magneto-inertial measurement unit in this study, we were unable to assess the effects of rehabilitation on step width, a well-known increased gait parameter in swCA attempting to compensate for balance impairment during gait [1, 7, 13, 15, 66, 67]. However, given the inverse correlation between step width and stride length, the correlation between ∆stride length and ∆sLLE_ML_ in this study may reflect a decrease in the need for compensating by widening the base of support due to improvements in trunk stability [20].

Moreover, pelvic rotation approached normative values after rehabilitation (Table 1, Fig. 2) and correlated with improvements in sLLE_AP_. Moreover, pelvic rotation improvements were significantly higher in swCA who improved their sLLE_ML_ by the ≥ 28.19% MCID (Table 4). Considering that impaired trunk-lower limb coordination [10], increased coactivation of lower limb muscles [18], and chaotic upper trunk behavior [68] may cause compensatory reductions in pelvic mobility during gait [36], it is possible to hypothesize that the proposed rehabilitation training, which included a large proportion of trunk stability-focused exercises, induced swCA to increase their pelvic movement due to improvements in trunk stability during gait.

However, useful biomarkers of gait instability and falls risk in swCA [36, 37], and HR and CV did not show responsiveness to rehabilitation in this study. HR represents a measure of the symmetry of acceleration signals reflecting gait symmetry or trunk smoothness during gait [56, 69], which has been reported as responsive to rehabilitation in subjects with Parkinson’s disease [35]. The results of this study lead us to hypothesize that, in swCA, the proposed intensive rehabilitation training may be more effective on trunk stability rather than trunk smoothness or symmetry as measured. However, because we measured the symmetry of trunk behavior during gait through HR only, we cannot exclude that other measures in the gait symmetry domain would have improved following rehabilitation. As regards the CV, a lack of reduction of variability in spatial gait parameters is expected in swCA. However, in this study, we only assessed the step-to-step variability. Conversely, Ilg et al. reported a reduction of the temporal variability in limb coordination during gait after rehabilitation [20–22]. Since we could not assess interlimb variability due to the use of a single lumbar-mounted magneto-inertial measurement unit, the responsiveness to the rehabilitation of other gait variability measures should be further investigated.

This study presents several limitations to be accounted for a better interpretation of the results. First, we only included swCA who could walk independently, excluding subjects with more severe disability, to which our findings cannot be directly extended. Additionally, because of the small sample size, we could not investigate differences between the subtypes of degenerative CA. Notably, we discovered that the three subjects who improved the most in SARA_GAIT_ also improved the most in sLLE. Despite the internal responsiveness of sLLE did not change substantially after removing these three subjects, we cannot rule out an overrepresentation of subjects with the greatest improvements in SARA_GAIT_ scores on sLLE improvements due to the small number of subjects who improved their SARA_GAIT_ scores by more than 1 point. As a result, our study deserves to be replicated with larger samples in order to better understand the factors that predict sLLE responsiveness after rehabilitation.

Furthermore, we only evaluated subjects with inherited degenerative CA; thus, our findings deserve to be confirmed on subjects with other cerebellar or afferent gait disorders. Although recommended in cerebellar disorders [45], another limitation of this study is represented by the use of SARA improvements as the criterion for external responsiveness calculations. Since a clear definition of responsiveness and minimal clinically important change scores for SARA are lacking [32, 70–72], in this study, we used the SDC scores as the external criterion. However, we cannot exclude that our results may vary by using other clinical scales as the criterion for clinical improvement definitions.

## Conclusions

In this study, we aimed at assessing the responsiveness of trunk acceleration-derived HR, sLLE, and CV, to a 4-week intensive rehabilitation program. sLLE in the ML and AP directions revealed good internal and external responsiveness, and moderately correlated with the improvements in SARA_GAIT_ subscore, stride length, and pelvic rotation. The findings of this study suggest that trunk stability can be effectively quantified using sLLE and improve after rehabilitation. Because of the usability and affordability of magneto-inertial measurement units, sLLE can be considered a useful additional outcome measure for assessing the effectiveness of intensive rehabilitation treatments, particularly when focusing on improvements in trunk stability during gait. Further studies including larger populations are needed to confirm these results and investigate long-term responsiveness.

### Supplementary Information

Below is the link to the electronic supplementary material.

Supplementary file1 (DOCX 17 KB)

## Data Availability

No datasets were generated or analysed during the current study.
