# Supplementary material for: Local Dynamic Stability of Trunk During Gait is Responsive to Rehabilitation in Subjects with Primary Degenerative Cerebellar Ataxia
Source: Cerebellum. 2024 Jan 27;23(4):1478–89. doi: 10.1007/s12311-024-01663-4 (PMC11269439; doi:10.1007/s12311-024-01663-4)
Supplement: Supplementary file 1 — Supplementary file1 (DOCX 17 KB) [file 12311_2024_1663_MOESM1_ESM.docx]

**Supplementary material**

**A. HRs, sLLE, and step length CV calculation**

| ***HRs calculation*** |
| --- |
| HR_AP_ and HR_V_ were calculated as the ratio of the sum of the amplitudes of the first 10 even harmonics to the sum of amplitudes of the first 10 odd harmonics, whereas HR_ML_ was calculated as the ratio of sum of the amplitudes of the first 10 odd harmonics to the sum of the amplitudes of the first 10 even harmonics, as follows:  ${HR}_{AP, V}=\frac{{\sum_{i} A}_{i*2}}{\sum_{i} A_{i*2-1}}$  ${{HR}_{ML}}= \frac{\sum_{i} A_{i*2-1}}{{\sum_{i} A}_{i*2}}$  where $A_{i}$ is the amplitude of the first 20 even harmonics and A2i–1 represents the amplitudes of the first 20 odd harmonics. The trunk accelerations of each stride were broken down into individual sinusoidal waveforms using a discrete Fourier transform. |
| ***sLLE calculation*** |
| The procedure described by Van Schooten et al. (2014) was used in this study to estimate sLLE [60]. To obtain 100 data points per stride, the accelerations were time - normalized. To avoid the loss of spatiotemporal fluctuations and nonlinearities, no filtering was applied to the accelerations, thereby excluding the effects of the length of the data series on LLE estimation. For each AP, ML, and V acceleration signal over the considered strides in each trial, the short-term maximum finite-time Lyapunov exponent (λ_max_) was computed using Rosenstein's algorithm for short time series and the Lyaprosen MATLAB toolbox for nonlinear time series analysis. A multidimensional state space was reconstructed from the recorded one-dimensional time-series data by juxtaposing the original data and delayed copies. The time delay was determined according to the first minimum of the AMI function, and the dimensions of the reconstructed space state were determined using the false nearest neighbor method. |
| ***CV calculation*** |
| CV was calculated as the ratio between the standard deviation and the mean stride length over all the stride lengths for each subject, as follows:  *CV*=100*SD/mean* |

**B. Abbreviations**

AP: Antero-posterior Direction of the Acceleration Signals

AUC: Area Under the ROC Curve

CV: Coefficient of Variation

HS: age and gait speed-matched Healthy Subjects

HR: Harmonic Ratio

LR+: Positive Likelihood Ratio

LR-: Negative Likelihood Ratio

MCID: Minimal Clinically Important Difference

ML: Mediolateral Direction of the Acceleration Signals

PTP+: Positive Post-test Probability

PTP-: Negative Post-test Probability

SAOA: Sporadic Adult-onset Ataxia

SARA: Scale for the Assessment and Rating of Ataxia

SARA_GAIT_: Gait Subscore of the SARA Scale

SCA: Spino - cerebellar Ataxia

Se: Sensitivity

sLLE: Short-term Largest Lyapunov Exponent

Sp: Specificity

swCA: subjects with primary degenerative Cerebellar Ataxia

V: Vertical Direction

∆ sLLE: Percentage Improvement of Short-term Longest Lyapunov’s Exponent Following Rehabilitation
